# Organizational Culture and Job Demands and Resources: Their Impact on Employees’ Wellbeing in a Multivariate Multilevel Model

**DOI:** 10.3390/ijerph16173006

**Published:** 2019-08-21

**Authors:** Esther Lopez-Martin, Gabriela Topa

**Affiliations:** 1 Department of Methods of Research and Diagnosis in Education II, Universidad Nacional de Educación a Distancia (UNED), Calle Juan del Rosal, 14, 28040 Madrid, Spain; 2 Department of Social and Organizational Psychology, Universidad Nacional de Educación a Distancia (UNED), Calle Juan del Rosal, 10, 28040 Madrid, Spain; 3 Faculty of Health Sciences, Universidad Politécnica y Artística del Paraguay, 1628 Asunción, Paraguay

**Keywords:** JDR theory, organizational culture, group and organizational identification, professional identity, job satisfaction, organizational citizenship behavior

## Abstract

(1) The present study aims to explore the impact of job demands and resources (JDR), personal resources, and the organizational culture on workers’ wellbeing and health. (2) A cross-sectional survey of Spanish workers in small and medium-sized enterprises (SMEs) was conducted with a sample of 1599 workers from 154 SMEs. A multivariate multilevel analysis was performed to analyze the different relationships. (3) In light of the results obtained, we observed that job demands were negatively associated with workers’ health, while job resources were positively correlated to workers’ health and wellbeing. Secondly, the different types of identification at work are positively related to job satisfaction and organizational citizenship behaviors (OCBs), but the intensity of this association differs with the form of identification. Finally, at the organization level, the dimensions of organizational culture are related differently to employees’ job satisfaction, OCBs, and health; (4) these results confirm the role of organizational culture and its association with desirable outcomes, allowing us to expand the JDR model.

## 1. Introduction

Positive organizational psychology has focused on promoting the wellbeing and health of people at work [1], based on a salutogenic model instead of a pathogenic one [2]. Psychological wellbeing consists of self-realization but also of social integration, contribution, and actualization, among other facets [3,4]. Hence, employees’ wellbeing can be conceptualized as hedonic or eudaimonic wellbeing. Hedonic wellbeing refers to the affective and cognitive components of satisfaction assessment, whereas eudaimonic wellbeing refers to the individual’s psychological and social functioning [5]. Following this approach, affective job satisfaction is considered a direct indicator of hedonic wellbeing at work, since it is linked to the presence of positive emotions and the absence of negative emotions elicited by the job itself [6].

However, from the eudaimonic perspective, wellbeing is connected to the social dimension. This would include a positive orientation of employees’ behaviors such as organizational citizenship behaviors (hereafter, OCBs) towards other people in the work environment or their organization. Along with the personal point of view, from an organizational perspective it is not sufficient for organizations just to have satisfied employees. They also need people who are involved in behaviors that are useful to the organization, but that exceed the requirements of the workplace. Health is a term that covers a broad spectrum of conditions. Self-reported health is a widely recognized indicator of personal wellbeing [7], and perceiving oneself as a healthy individual is considered a good predictor of lifespan and full participation in society. 

The theory of job demands and resources (JDR) has enriched our understanding of the bases of workers’ health and wellbeing [1]. However, most empirical studies in the literature have studied the importance of job demands and resources in relation to the work post [8], paying little heed to the fact that workers are integrated within the organizations and these demands and resources are characterized by a culture. The organizational culture is a set of values, rules, and beliefs shared by its members, which create an internal environment that is taught to new members. This culture can, therefore, impose internal contextual factors that act as boundary conditions for the influence of job demands and resources on employees’ outcomes [9]. Hence, it is crucial to integrate predictors from other levels into the model to explain how the influence of job demands and resources on workers’ health and wellbeing is affected by the organizational culture.

A promising extension of the JDR theory includes personal resources that can enhance employees’ wellbeing [10]. These include workers’ beliefs about having some control over their environment, and there is evidence that some personal resources, such as organization-based self-esteem, have a positive effect on attitudes and behaviors of work engagement [11]. However, there are few studies in the literature about this type of resource. Moreover, none of the published studies tried identifying workers with different foci (resulting from their integration in different entities—group, profession, and organization [12]—) or personal resources to analyze their potential effects on workers’ health and wellbeing.

To sum up, empirical research into the factors involved in employees’ wellbeing and health is extensive, and the influence of job demands and resources has been a major focus [8]. Moreover, personal resources, such as identification-based self-esteem, and the relationships between wellbeing and identity, have been documented [10]. Lastly, empirical research supports a probable influence of organizational culture on employee wellbeing and health [13,14].

Hence, in this study we propose that identification with different foci plays the role of a personal resource, as it constitutes a measure of self-esteem based on group membership, profession, and the organization [12], and will be positively related to employee wellbeing and health [11]. The integration of these two aspects aims to more accurately account for the multiple associations present in the work setting [15]. There, demands and job resources, personal resources based on group membership, profession, organization, and values and shared standards are related to workers’ results. In response to some criticism [16], this study helps us to understand how the associations between demands, resources, and outcomes vary depending on the employee’s relationship with different foci, and how they are contextualized within the organizational culture. It will therefore be unnecessary to resort to other theories to explain the underlying psychological processes, as [17] points out, and practical interventions aimed at increasing employees’ welfare can be proposed.

### 1.1. Job Demands and Resources

Job demands include the physical, psychological, organizational, and social aspects that require employees’ efforts and are associated with the physical or psychological costs [18] of them performing their work. Resources refer to those aspects of work that contribute to goal achievement, reduction of demands and their associated costs, or to personal growth and development [19].

The link between demands and resources and personal and organizational outcomes involves two processes [20,21]. On the one hand, demands initiate health impairment processes often related to undesirable outcomes, such as exhaustion or health problems. On the contrary, resources are often associated with motivational processes that have an impact on desirable outcomes, such as job satisfaction and citizenship behaviors [22]. Abundant empirical research and meta-analytical reviews support the predictive power of demands and resources on employees’ wellbeing and health (e.g., [23,24,25,26,27,28,29]). Therefore, the present study proposes that workers’ perception of job demands and available resources is associated with job satisfaction, their OCBs and health (Hypothesis 1).

### 1.2. Identification with Different Foci as a Personal Resource

Personal resources play a similar role to job resources and the part played by organization-based self-esteem [15], among other factors, has been analyzed. Given that social identification is the part of self-conception derived from belonging to different social groups, it is linked to the emotional and evaluative meanings attached to that membership [30]. Therefore, identification involves cognitive and evaluative components associated with the integration of one’s own group, profession, or organization in the self-concept.

Although organizational identification was initially considered the most relevant form of social identity in work settings [31], more recent studies have indicated that workers develop multiple identifications with different foci that are available to them [13]. In this line, the role of identification with the group has been explored [32], and subsequently, with the profession as a more localized, transportable focus of identification, which, to some extent, is more under the employee’s control [33,34].

A literature review also enabled us to find some studies that related these types of identification at work with different aspects of workers’ wellbeing, such as job satisfaction, the willingness to work for the group’s interests, or their health. Firstly, regarding job satisfaction, a recent meta-analysis [35] suggested that this is usually a result of identification, because it seems to lead to people taking on the defining characteristics of the group with which they identify. Thus, through identification, the group, profession, or organization becomes a part of the self [32] and, as people tend to positively assess the objects associated with their self, they are expected to develop feelings of satisfaction towards their group, profession, or organization.

Secondly, it has been clearly established that, as people become more closely identified, they are more likely to act in accordance with a social identity implied by membership to a group [36]. This also implies that people are willing to adopt the views of the group. Even if they oppose group interests, they will set aside their own personal interests in order to pursue the group’s. Thus, identification is associated with many desirable outcomes in organizations, such as extra-role behavior, contextual performance, and OCBs [37].

Thirdly, a growing body of empirical evidence supports the positive influence of social identification on health [38,39]. These works confirm that identification provides meaning to life and generates a sense of belonging, which are antithetical processes to illness [40], although none of these tested for possible identification with different foci at work.

In spite of the importance of these findings, more empirical research is required to study the influence of different types of identification at work (with the group, profession, and organization) on workers’ wellbeing and health. Therefore, this study proposes that these types of identification at work, considered as personal resources, are related to workers’ job satisfaction, their OCBs, and health (Hypothesis 2).

However, while it is true that all these forms of social identity at work have shown their influence on outcomes, the relative importance of each of them is under debate, as empirical studies have found these identifications to have directional effects on the results [36,41]. In accordance with the principle of consistency between attitude and behavior, we suggest that the prediction of behavior from attitude will be more effective if both are considered at the same level of specificity. In this sense, meta-analytical findings have verified that group identification is more strongly linked to outcomes focused on the group, whereas organizational identification is more closely associated with outcomes referring to the organization as a whole. The same pattern of relationships between predictors and criteria is weakened when some variables belong to the group level and others to the organizational level [37]. Nevertheless, in the aforementioned meta-analysis, professional identification was not considered and, as in most primary studies, results that could effectively be predicted from this kind of identification at work are not proposed.

Therefore, together with the previous hypothesis, this study proposes that the magnitudes of relationships between the different forms of identification at work (with the group, profession, and organization) and the employees’ results will be different (Hypothesis 3). Due to the scarcity of previous literature, it is not possible to formulate more specific hypotheses about the relationships between identification with each foci and employees’ job satisfaction, OCBs, and health.

### 1.3. Organizational Culture

Organizational culture is a set of core values, assumptions, understandings, and norms that is shared by the members of an organization and proposed to new members as correct [42]. These shared norms, values, and beliefs generate an environment that has an apparent influence on employees’ attitudes and behaviors [11,12,43,44,45]. However, although organizational culture has been linked to employees’ performance and an organization’s effectiveness for decades [46], the influence of organizational culture on the relationship between job demands and resources and employees’ outcomes has received less attention. Although some empirical studies have shown how an organizational-level safety climate can explain the origins of job demands and resources, and influences employees’ health and engagement at the lower level [47], in general, as pointed out by Schaufeli and Taris [16], the job demands-resources (JDR) theory has paid scant attention to the combination of predictors at different levels.

Even though some empirical studies that have examined the relationship between JDR and employees’ outcomes have taken organizational features into account, most research has focused on specific organizational features, such as ethical culture [48] or culture error management [49], without considering the more general features shared by a wider range of organizations.

Instead, in this study, organizational culture is considered based on the competing values model [50]. Hence, four different dimensions of organizational culture are defined and assessed: support, innovation, rules-, and goal-oriented [51]. We suggest that each of these dimensions has a different relationship with employees’ wellbeing, in particular with perceived health, job satisfaction, and OCBs (Hypothesis 4). The role of organizational culture in the relationships between JDR and employees’ outcomes is still unclear, and there is a lack of empirical evidence to suggest more specific hypotheses.

**Figure 1:**
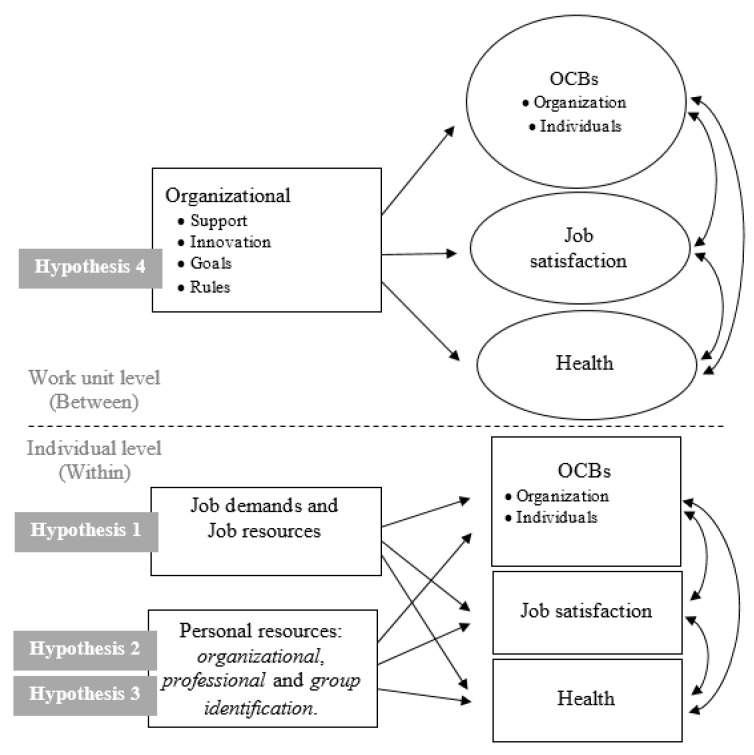
Multivariate multilevel model. OCBs = Organizational citizenship behaviors.

Figure 1 presents the proposed research model and the four hypotheses underlying it.

## 2. Materials and Methods

### 2.1. Participants

The data come from a quantitative study using a survey of workers in small and medium-sized enterprises (SMEs—that is, enterprises with fewer than 249 workers) in Spain, carried out during the months of June and December 2016. In January of that year, there was a total of 3,228,747 SMEs in Spain, of which 55.45% were companies with no paid employees, which were consequently excluded from the study. The number of workers in the remaining SMEs ranged between one and nine for 40.37% of the enterprises, between 10 and 50 for 3.58%, and between 50 and 249 workers for 0.60% [52]. The sample was made up of 1599 workers from 154 SMEs, and the average number of employees per company was 10. Regarding the age of the sample, 7.5% of these workers were younger than 25, 33.2% were between 25 and 35 years, 34.6% were between 35 and 45, 17.5% were between 45 and 55, and 7.1% were older than 55 years old. Regarding gender, 49% of the participants were men. As to educational level, 9.2% of the subjects had basic studies, 38.2% had a high school or vocational training degree, and 52.6% had completed higher studies. The professional status was divided into 8.1% unskilled workers, 75% in an administrative or technical post, 13.1% middle managers, and 3.9% managers. Concerning tenure, 15.2% of the sample had been working in the organization for less than one year, 49% had been working for over one year but less than 10 years, 22.7% had been working between 10 and 20 years, 9.5% between 20 and 30 years, and 3.6% for over 30 years. Finally, 83% of the sample worked full time, 15.5% part time, and about 1.5% had a different job situation.

### 2.2. Procedure

In the first stage of sample selection, incidental non-probability sampling was performed, from which we selected 154 SMEs. In a second phase, the workers in these companies were informed of the purpose of the research and guaranteed anonymity and confidentiality for their responses. Information was collected through a paper-and-pencil version of the questionnaire that was distributed around the companies that agreed to participate in the study. Participants also received an envelope in which they placed the completed questionnaires, which were subsequently collected from their company by the research team.

### 2.3. Ethical Statement

The Bio-ethical Committee of the National Distance Education University (UNED) approved the project in May 2016.

### 2.4. Instruments

*Job demands* were evaluated by the job demands subscale of the job content questionnaire (JCQ) [53], validated in its Spanish version by Escribà-Agüir, Más Pons, and Flores Reus [54]. The items assess the amount of work that must be done in that job, the speed required when performing it, the complexity of the tasks, and the intensity with which the work must be performed. Examples of items are: “My job requires me to work very quickly”, “My job requires me to work hard”, “I am asked to do an excessive amount of work”, or “My work requires long periods of intense concentration”. The study of the adaptation to Spanish yielded a reliability of 0.74 for this version. Cronbach’s alpha in this study was 0.75.

*Job resources* were evaluated using the control and support subscales of the JCQ, in the Spanish version validated by Escribà-Agüir and collaborators [53]. Job characteristics (the possibility of controlling the work, the use of competencies, the development of competences in the post, and the professional and emotional support received) were evaluated with 15 items. Examples of these items are: “My job often allows me to make my own decisions”, “My job requires a high level of competence”, “In my work, I perform varied activities”, or “I have the opportunity to develop my professional skills”. The original validation study found adequate reliability, ranging between 0.75 and 0.84. Cronbach’s alpha in this study was 0.85.

*Personal resources* refer to organizational identification, professional identification, and group identification. Following the procedure used in other studies [16,55], identification with each focus was measured with five items from Mael and Ashforth’s identification scale [31]. We replaced the referent of the items to represent the respective foci (i.e., the team, profession, or organization). Sample items are “The successes of my organization/team/profession are my successes” and “When I talk about my organization/team/profession, I usually say ‘we’ rather than ‘they’”. Cronbach’s alpha values in the present study for each subscale were: 0.78 for organizational identification, 0.73 for professional identification, and 0.77 for group identification.

*Organizational culture*. We used the Spanish version [56] of the FOCUS 93 questionnaire [51], which assesses the frequency of certain features of one’s own organization. The underlying cultural model of this questionnaire was Quinn and Rohrbaugh’s [50] competing values model. Based on two bidimensional axes (internal vs. external orientation and flexibility vs. control), the survey provides four organizational culture orientations: support, innovation, rules, and goals. Key concepts for support orientation are participation, cooperation, mutual trust, and team spirit. The innovation is characterized by the search for new information, creativity, openness to change, and anticipation. The rules emphasize respect for authority, rationality of the procedures, and the division of work. Goal orientation emphasizes performance indicators, accomplishment, and accountability [51]. Support orientation includes eight items, innovation 12 items, rules six items, and goals 14 items. Examples of items are “How many people with personal problems are helped?”, “How often is constructive criticism accepted?” (support), “How often does your organization search for new markets for existing products?” (innovation), “How often are instructions written down?” (rules), and “How often is it clear how performance will be evaluated?” (goals). Reliability values in the original study were 0.91 for support, 0.69 for innovation, 0.77 for rules, and 0.76 for goals. In this study, Cronbach’s alpha values for each subscale are: 0.84 for support, 0.86 for innovation, 0.87 for rules, and 0.66 for goals.

*Job Satisfaction*. The brief index of affective job satisfaction (BIAJS, [6]) was used. This brief scale is a measure of affective job satisfaction with just one factor composed of four items: “I find real enjoyment in my job”, “I like my job better than the average person”, “Most days I am enthusiastic about my job”, and “I feel fairly well satisfied with my job”. Responses are rated on a five-point Likert scale ranging from 1(strongly disagree) to 5 (strongly agree). Moreover, in order to reduce priming effects and acquiescent responses, the scale includes three distracter items: “My job is unusual” (between items 1 and 2), “My job needs me to be fit” (between items 2 and 3), and “My job is time-consuming (between items 3 and 4). In this study, the Cronbach’s alpha of this scale is 0.88.

*Organizational Citizenship Behavior*. We used a Spanish adaptation [57] of the organizational citizenship behavior scale designed by Lee and Allen [58]. This 16-item instrument has the advantage of evaluating both OCBs directed at the organization (OCB_O_) and OCBs directed at individuals (OCB_I_), unlike other scales that only focus on the perspective of the institution. Therefore, it provides two separate, but correlated measures of the behaviors aimed at benefiting peers and companies. Subjects report how often they perform the actions described in the items at work on a five-point Likert-type response format ranging from 1 (infrequently) to 5 (frequently). Example items are: “I show an interest in the organization’s image” and “I dedicate time to helping others who have problems related or not to the tasks”. The reliability analysis showed satisfactory internal consistency for the scale in previous studies [59]. In our sample, the Cronbach’s alpha value of the OCB_O_ subscale is 0.90 and of the OCB_I_ subscale it is 0.87.

*Health*. Self-rated health status was assessed with four items, following the procedure of the survey of health, aging, and retirement in Europe (SHARE) [60]. Questions from the self-completion questionnaire (nr. 4, options a, d, m, and l) were included, two of which assess depression and exhaustion and the other two happiness and high energy. The first two items were reversed so as to indicate a global measure of good health. The Cronbach’s alpha of this scale was 0.70 in this study.

All the scales were responded on a Likert-type response scale ranging from 1 (totally disagree/never/infrequently) to 5 (totally agree/always/frequently).

Along with the questions relating to these scales, information was gathered on the following sociodemographic characteristics, which are the control variables in our model: age (0: younger than 25 years; 1: between 25 and 35; 2: between 35 and 45; 3: between 45 and 55; 5: over 55 years), gender (0: men; 1: women), education (0: basic studies; 1: high school degree or vocational training; 2: higher education), professional category (0: unskilled worker; 1: administrative staff or technician; 2: middle management; 3: manager), tenure (0: less than one year; 1: between 1 and 10 years; 2: between 10 and 20 years; 3: between 20 and 30 years; 4: over 30 years), and work situation (1: full-time and 2: part-time).

### 2.5. Data Analysis

Firstly, the participants’ scores in each of the scales were calculated. For this purpose, we applied the generalized partial credit model (GPCM) [61], developed under the assumptions of the item response theory [62] for situations with ordinal items. The scores were then transformed to a scale with a mean of 250 and standard deviation of 25.

In the second phase, we estimated the multivariate multilevel model [63], in which all four response indicators were entered at once: (1) job satisfaction, (2) OCB_O_, (3) OCB_I_, and (4) health. We specified a two-level model, in which the employees (level 1) are grouped into organizations (level 2).

The Mplus version 8 (Muthén & Muthén, Los Angeles, CA, USA) [64] program was used to estimate the models and, to facilitate the interpretation of results, the quantitative predictors on workers’ level were group-mean centered, and the predictors on organization’s level were grand-mean centered. Regarding the sociodemographic characteristics, “male,” in the case of gender, and the initial categories of the ordinal variables (age, level of studies, professional category, and tenure) were taken as reference categories. Finally, to identify the workers’ job situation, we created two dummy variables (full-time and part-time).

#### Data Aggregation

Given that the dimensions of organizational culture were measured at the worker level, we estimated the mean value of the organization in these indicators in order to analyze its relationship with the wellbeing perceived by employees of different organizations. Analysis of the data’s adequacy to be aggregated was carried out by evaluating the inter-rater agreement (IRA) and intraclass correlation. Thus, we calculated the within-group interrater reliability RWG (j) index for multiple item scales [65], the average deviation (AD) index [66], and the intra-class correlation coefficients (ICC_(1)_ and ICC_(K)_).

The results show that, except for two organizations (RWG_(36)_ = 0.49 and RWG_(148)_ = −0.05), the RWG (j), values for the predictor support were higher than 0.70, and higher than 0.80 for 96% of the SMEs. Similar results were observed for the other predictors: innovation (all the values exceeded 0.79), rules (approximately 4.5% of the organizations obtained values between 0.7 and 0.8, obtaining higher values in the remaining cases), and goals (all the organizations presented values higher than 0.72, and in 98% of the companies, higher than 0.83). The above values revealed a strong measure of agreement among the workers [67].

A similar trend was observed when analyzing the AD indices. In the case of predictor support, 91.56% of the organizations did not exceed the critical value suggested by Burke and Dunlap [68] for five-point Likert-type response scales, that is, 0.83 (c/6 = 5/6). For the predictor’s innovation, rules, and goals, these values were, respectively, 93.51%, 94.81%, and 90.26%. We note that only three organizations in the support dimension (AD_(100)_ = 1.03, AD_(36)_ = 1.11, and AD_(148)_ = 1.19) and one in the goal dimension (AD_(36)_ = 1.03) presented AD indices higher than 1 [66].

According to the intra-class correlation coefficients, the ICC_(1)_ values were higher than 0.37 in all cases for the predictors support, innovation, and goals, and higher than 0.36 for the dimension rules, so they could all be considered as appropriate (higher than 0.25). The ICC_(K)_ values were higher than 0.83 in all cases.

Considering the calculated indices and coefficients, the data’s adequacy to be aggregated was justified. This aggregation was performed for all the organizations and indicators analyzed, apart from two companies in which unsatisfactorily low levels of agreement were observed, both in the Rwg index and the AD index. These two organizations were omitted from the analysis, so the final sample was composed of 1579 workers from 152 SMEs.

## 3. Results

Table 1 and Table 2 present the main descriptive statistics for the response variables and level 1 (employee) and level 2 (organization) predictors entered in the model. They also present the bivariate correlation matrices between the different scales.

Table 3 presents the fixed part of the model that informs about the effect of job demands and resources, personal resources, and the dimensions of the organizational culture on employee’s satisfaction, OCBs, and health, and of the mean value in each of the response variables after controlling for the effect of level 1 and 2 predictors.

In light of the results obtained, and regarding the first research hypothesis we formulated, we observed that job demands were negatively associated with employees’ perception of health, whereas job resources are positively associated with all the variables considered.

Regarding personal resources, the second research hypothesis was partly confirmed, as the different forms of identification at work could predict job satisfaction and OCBs but not employees’ health. At the same time, the results supported the third working hypothesis, which proposed a differential effect of the types of identification at work on employees’ outcomes.

In relation to the organization-level predictors, the dimensions of organizational culture are related differently to employees’ job satisfaction, OCBs, and health (Hypothesis 4). We observed that employees in organizations with a support-oriented culture are more satisfied with their jobs and have more behaviors aimed at benefiting peers and companies (OCBs). Also, the innovation orientation favors OCBs, but only those that are oriented towards the other individuals of the organization. Moreover, workers in organizations with a goal-oriented culture perceive their health as worse than average. However, if the organizational culture is oriented toward rules, employees report a better health status.

Finally, we refer to the effect of the different sociodemographic characteristics considered. Thus, the results revealed a positive effect of the level of studies on job satisfaction and OCBs oriented to people, and of the professional category on OCBs oriented to the organization. The perception of health seems to be negatively affected by employees’ gender (being a woman) and age (being older).

The random part of the model is presented in Table 4. It should be noted that, along with the random parameters of this model, there are those relating to the null model—that is, the model that does not include explanatory variables at any of its levels. The comparison of the two models allows us to evaluate the fit of the model. Considering a difference in deviances of 3463.36, with 64 degrees of freedom, the associated probability would be equal to 0.000. This implies that, after entering the predictors, the resulting model (contextualized model) fits the data significantly better than the null model. In the same way, the values of Akaike’s information criterion (AIC) and the Bayesian information criterion (BIC) were lower in the case of the model with predictors, so this model is more appropriate to explain workers’ wellbeing, job satisfaction, and health.

For each of the levels, the residual variances in the different response variables and the covariances between them are presented. The estimated random parameters reflect the existence of unexplained variance both at the employee level and the organization level, implying a variability in the employees’ perception of wellbeing and health within the same organization and in the different SMEs considered. The estimated covariances and their corresponding correlations, shown in Table 5, show a positive association between all the response variables at the employee level. At the organization level, the intensity of this relationship increases in some cases, but not in all of them. In this regard, in the contextualized model, only the covariances between satisfaction and OCB_O_ and between OCB_I_ and OCB_O_ were significant.

The comparison between the random parameters of the two models shows that entering the sociodemographic characteristics and other predictors relating to the employees and the organization led to a significant reduction in the unexplained variance. Table 6 reflects the proportion of variance explained after entering the predictors in the model.

These predictors explain approximately 25% of the difference between employees and 45% of the variance between organizations. The response variables considered help to explain an important variance in satisfaction (40.675%) and in OCB_O_ (42.500%), and, to a lesser extent, the differences in OCB_I_ (25.788%) and in the perception of health (16.036%).

## 4. Conclusions

The present study provides evidence of the relationship between different foci at work, such as personal resources, job satisfaction, OCBs, and employees’ perception of their health. It also confirms the role of the organizational culture and its association with desirable outcomes, allowing us to expand the JDR model. These are important contributions to the JDR theory, which has been criticized [16] for its need to constantly rely on other theories to explain the psychological processes underlying its results.

So far, the literature has focused on understanding the relationships between demands and resources to explain employees’ wellbeing, with scarce evidence that this wellbeing is influenced by employee identification with different foci and is contextualized in the organizational culture. Discovering some of the complex relationships between demands, resources, identification, and organizational culture, the present study seeks to improve our understanding of the different patterns of associations between demands and resources as a function of the group and organizational context. The positive relationship between identification with different foci at work, understood as a source of self-esteem, and the impact of certain dimensions of the organizational culture on wellbeing are consistent with the JDR model and the social identity theory applied to organizations [69].

Relying on the principle of compatibility between attitudes and behaviors, we hypothesize that the different foci of identification will have different predictive capacities for job satisfaction and OCBs oriented to the organization or to individuals. The findings confirm that, whereas people who are more identified with their profession and organization feel more satisfied and present more OCBs oriented to their organization, workers who identified more with the work group are more likely to perform OCBs directed towards other people.

It is true that investigation of the JDR model has expanded in recent years to include personal resources [70], but these have been conceptualized mainly as optimism [71] or self-esteem, and, therefore, have not progressed sufficiently regarding the design of strategies to increase such resources [72]. Our findings, however, suggest that the groups, organizations, and even the profession can play an active role in increasing workers’ arsenal of resources to cope with job demands. Thus, the present evidence extends the debate about personal resources and opens up a promising interventional approach aimed at achieving specific outcomes at employees’ attitudinal and behavioral levels.

Consistent with the proposed model, the organizational culture is confirmed as a construct of higher order, the dimensions of which are related differently to employees’ satisfaction and OCB’s. Thus, the dimensions of support and innovation appear to be related to employees’ attitudes and behaviors, whereas goals and rules have an impact on health. However, and more important still, it seems that a goal-oriented culture, probably due to its emphasis on competitiveness and the achievement of results, is detrimental to employees’ health, whereas rule orientation, probably because it provides an environment of predictability and certainty, has a positive impact on employees’ health. These associations had not been clearly established before. In addition, an important proportion of variance can be explained after introducing predictors in the model, especially variance between organizations. Therefore, the most important contribution of this work is responding to the reminder of Bakker and Demerouti [15] about the need to consider the multilevel nature of the data and to investigate the perceptions shared by employees in the units, departments, and companies. Adoption of a multilevel approach has respected the nested data structure and enabled possible differences in the wellbeing of workers to be studied in relation to the different levels of grouping. In turn, a multivariate model can analyze the different indicators of wellbeing together.

Finally, although the differences between males and females are not the subject of the present research, the results show that women have a worse perception of their own health than men. These findings appear to support recent evidence about gender differences in the JDR model [73,74], which should be explored in the future [75].

### Limitations and Future Directions

The limitations of this research include the cross-sectional nature of the data, which does not allow us to establish causality between the variables included in the study. A second limitation is that, although variables at different levels are included in the model, other variables have not been evaluated at the level of the team, such as team cohesion or team climate, which could affect the results. Longitudinal studies affecting team measures are necessary to account for the interactions between the resources at different levels and the possibility of reverse causation between outcomes and demands [76].

Because it provides evidence about the promoter role of identification in employees’ wellbeing, this work offers organizations a clear strategy to gain a competitive advantage, because satisfaction and extra-role behaviors can increase through interventions that foster group, professional, and organizational identity. Supervisors should be trained to monitor and strengthen the characteristics of organizational culture that promote wellbeing, and to manage and provide support to workers when coping with characteristics of the culture that can have harmful effects on their health. Practical implications are related to the positive effects of identification with different foci at work, but also to the diverse characteristics of organizational culture. In detail, the present study suggests that workers’ job satisfaction and OCBs appear to be related to a support- and innovation-oriented culture. Hence, organizations should be aware of the potential impact of organizational culture features as a tool for promoting extra-role performance and worker satisfaction. Consequently, they should foment interventions that foster support and innovation as ways to increase structural resources and challenge demands, which have been proposed as two influential components in the job satisfaction boosting process [17].

Our results are not just interesting for firms, but also have implications for public health policy. Considering that our findings suggest that workers’ health seems to be favored by a rules-oriented culture, interventions focused on increasing role clarity and reducing ambiguity could be considered powerful contributors to creating healthier working environments.

In conclusion, this study provides broad support to the role of the foci of identification at work and the characteristics of organizational culture within the JDR model. Nonetheless, it is important to continue expanding the model by including other levels, such as the team, which are often not considered, and integrating constructs from other levels that can provide additional information to help design more effective interventions [77].

## Figures and Tables

**Table 1:** Means, standard deviations, and employee-level correlations (*n* = 1579).

Scales	Mean	SD	2	3	4	5	6	7	8	9
1. Satisfaction	250.157	23.097	0.369 **	0.575 **	0.401 **	−0.121 **	0.549 **	0.536 **	0.520 **	0.434 **
2. OCB_I_	250.295	23.131	-	0.574 **	0.204 **	0.026	0.458 **	0.268 **	0.348 **	0.406 **
3. OCB_O_	250.163	23.378		-	0.303 **	−0.026	0.537 **	0.456 **	0.565 **	0.482 **
4. Health	250.067	21.275			-	−0.174 **	0.305 **	0.207 **	0.210 **	0.181 **
5. Demands	250.127	22.599				-	−0.251 **	0.063 *	−0.009	0.048
6. Resources	250.244	23.496					-	0.322 **	0.454 **	0.479 **
7. Professional Identification	250.219	21.086						-	0.715 **	0.666 **
8. Organizational Identification	250.159	21.906							-	0.782 **
9. Group Identification	250.090	21.589								-

SD = standard deviation; OCB_O_ = OCBs directed at the organization; OCB_I_ = OCBs directed at individuals; ** *p* < 0.01.

**Table 2:** Means, standard deviations, and organization-level correlations (*n* = 152).

Scales	Mean	SD	2	3	4
1. Support	249.961	17.954	0.657 **	0.544 **	0.505 **
2. Innovation	250.148	19.716	-	0.597 **	0.729 **
3. Rules	250.084	18.715		-	0.630 **
4. Goals	250.363	15.044			-

SD = standard deviation; ** *p* < 0.01.

**Table 3:** Fixed part of the model.

Parameters	Satisfaction		OCB_I_		OCB_O_		Health	
	Estimate	S.E.	Estimate	S.E.	Estimate	S.E.	Estimate	S.E.
Intercept	253.396 **	7.517	237.311 **	7.719	233.869 **	6.777	256.888 **	7.286
**Employee-Level Predictors**								
Age							−1.483 **	0.504
Gender							−3.645 **	0.963
Educational level	2.037 **	0.740	2.217 *	0.916				
Professional category					4.373 **	0.981		
Years of experience								
Full Time								
Part Time								
Demands							−0.135 **	0.037
Resources	0.346 **	0.039	0.224 **	0.042	0.290 **	0.035	0.262 **	0.041
Professional identification	0.314 **	0.040			0.153 **	0.042		
Organizational identification	0.154 **	0.044			0.248 **			
Work group identification			0.197 **	0.048		0.041		
**Organization-Level Predictors**								
Support	0.579 **	0.083	0.312 **	0.070	0.559 **	0.080		
Innovation			0.239 **	0.077				
Goals							−0.180 *	0.092
Rules							0.339 **	0.090

S.E. = standard error; * *p* < 0.05; ** *p* < 0.01; Only significant parameters are included.

**Table 4:** Random part of the null model and the contextualized model.

Parameters	Null Model				Contextualized Model			
	Employee-Level		Organization-Level		Employee-Level		Organization-Level	
	Estimate	S.E.	Estimate	S.E.	Estimate	S.E.	Estimate	S.E.
Satisfaction variance	338.498 **	21.162	195.447 **	31.634	212.017 **	11.647	104.747 **	15.403
OCB_I_ variance	379.778 **	22.172	159.147 **	28.404	314.116 **	17.588	85.833 **	13.439
OCB_O_ variance	310.226 **	20.819	236.589 **	37.299	205.328 **	12.615	109.091 **	17.788
Health variance	346.160 **	19.045	107.729 **	19.823	296.790 **	16.163	84.313 **	13.925
Satisfaction and OCB_I_ covariance	122.456 **	14.626	77.193 **	22.024	43.020 **	8.398		
Satisfaction and OCB_O_ covariance	169.832 **	15.654	142.336 **	33.704	61.034 **	7.147	44.267 **	15.405
Satisfaction and health covariance	153.576 **	13.214	43.723 *	20.437	81.278 **	8.119		
OCB_I_ and OCB_O_ covariance	192.441 **	16.634	120.072 **	24.581	121.033 **	11.056	30.586 **	10.242
OCB_I_ and health covariance	79.323 **	11.807			35.373 **	9.727		
OCB_O_ and health covariance	98.527 **	10.675	52.623 *	22.352	39.676 **	8.073		
AIC	54,401.765				51,066.403			
BIC (Adjusted)	54,530.514				51,255.174			
Deviance	54,353.764				50,890.404			
Number of parameters	24				88			

S.E. = standard error; * *p* < 0.05; ** *p* < 0.01; Only significant parameters are included.

**Table 5:** Correlation between the random model parameters.

Variances	Null Model			Model Predictors		
**Employee-Level**	**2**	**3**	**4**	**2**	**3**	**4**
1. Satisfaction	0.342	0.524	0.449	0.167	0.293	0.324
2. OCB_I_	1	0.561	0.219	1	0.477	0.116
3. OCB_O_		1	0.301		1	0.161
4. Health			1			1
**Organization-level**	**2**	**3**	**4**	**2**	**3**	**4**
1. Satisfaction	0.438	0.662	0.301		0.414	
2. OCB_I_	1	0.619		1	0.316	
3. OCB_O_		1	0.330		1	
4. Health			1			1

**Table 6:** Explained variance.

Explained Variance (*R*^2^)	
Employee-Level	25.200%
Organization-level	45.060%
Criterion variable: Satisfaction	40.675%
Criterion variable: OCB_I_	25.788%
Criterion variable: OCB_O_	42.500%
Criterion variable: Health	16.036%
